# Supplementary material for: HIVprotI: an integrated web based platform for prediction and design of HIV proteins inhibitors
Source: J Cheminform. 2018 Mar 9;10:12. doi: 10.1186/s13321-018-0266-y (PMC5845081; doi:10.1186/s13321-018-0266-y)
Supplement: Supplementary file 2 — Additional file 2. Source code of HIVProtI web server. [file 13321_2018_266_MOESM2_ESM.zip › HIVprotI_Source-code/hivprotI/weka-3-6-6/doc/weka/attributeSelection/ASEvaluation.html]

ASEvaluation


---


|  |  |  |
| --- | --- | --- |
| **Overview** \| **Package** \| **Class** \| **Tree** \| **Deprecated** \| **Index** \| **Help** \| **Weka's home** | |  |
| PREV CLASS   **NEXT CLASS** | **FRAMES**    **NO FRAMES**     **All Classes** |  |
| SUMMARY: NESTED \| FIELD \| CONSTR \| METHOD | DETAIL: FIELD \| CONSTR \| METHOD |  |


---


## weka.attributeSelection Class ASEvaluation

```
java.lang.Object
  weka.attributeSelection.ASEvaluation
```

**All Implemented Interfaces:**: java.io.Serializable, CapabilitiesHandler, RevisionHandler

**Direct Known Subclasses:**: AttributeSetEvaluator, CfsSubsetEval, ChiSquaredAttributeEval, ConsistencySubsetEval, CostSensitiveASEvaluation, FilteredAttributeEval, FilteredSubsetEval, GainRatioAttributeEval, HoldOutSubsetEvaluator, InfoGainAttributeEval, OneRAttributeEval, ReliefFAttributeEval, SVMAttributeEval, SymmetricalUncertAttributeEval, UnsupervisedAttributeEvaluator, UnsupervisedSubsetEvaluator, WrapperSubsetEval

---

``` public abstract class ASEvaluation extends java.lang.Object implements java.io.Serializable, CapabilitiesHandler, RevisionHandler ```

Abstract attribute selection evaluation class

**Version:**
:   $Revision: 5510 $

**Author:**
:   Mark Hall (mhall@cs.waikato.ac.nz)

**See Also:**: Serialized Form

---

**Constructor Summary**

`ASEvaluation()`


| **Method Summary** | |
| --- | --- |
| `abstract  void` | `buildEvaluator(Instances data)`             Generates a attribute evaluator. |
| `static ASEvaluation` | `forName(java.lang.String evaluatorName, java.lang.String[] options)`             Creates a new instance of an attribute/subset evaluator given it's class name and (optional) arguments to pass to it's setOptions method. |
| `Capabilities` | `getCapabilities()`             Returns the capabilities of this evaluator. |
| `java.lang.String` | `getRevision()`             Returns the revision string. |
| `static ASEvaluation[]` | `makeCopies(ASEvaluation model, int num)`             Creates copies of the current evaluator. |
| `int[]` | `postProcess(int[] attributeSet)`             Provides a chance for a attribute evaluator to do any special post processing of the selected attribute set. |

**Methods inherited from class java.lang.Object**

`equals, getClass, hashCode, notify, notifyAll, toString, wait, wait, wait`

**Constructor Detail**

### ASEvaluation

```
public ASEvaluation()
```


**Method Detail**

### buildEvaluator

```
public abstract void buildEvaluator(Instances data)
                             throws java.lang.Exception
```

:   Generates a attribute evaluator. Has to initialize all fields of the
    evaluator that are not being set via options.

    :   **Parameters:**: `data` - set of instances serving as training data **Throws:**: `java.lang.Exception` - if the evaluator has not been generated successfully

---


### postProcess

```
public int[] postProcess(int[] attributeSet)
                  throws java.lang.Exception
```

:   Provides a chance for a attribute evaluator to do any special
    post processing of the selected attribute set.

    :   **Parameters:**: `attributeSet` - the set of attributes found by the search **Returns:**: a possibly ranked list of postprocessed attributes **Throws:**: `java.lang.Exception` - if postprocessing fails for some reason

---


### forName

```
public static ASEvaluation forName(java.lang.String evaluatorName,
                                   java.lang.String[] options)
                            throws java.lang.Exception
```

:   Creates a new instance of an attribute/subset evaluator
    given it's class name and
    (optional) arguments to pass to it's setOptions method. If the
    evaluator implements OptionHandler and the options parameter is
    non-null, the evaluator will have it's options set.

    :   **Parameters:**: `evaluatorName` - the fully qualified class name of the evaluator: `options` - an array of options suitable for passing to setOptions. May be null. **Returns:**: the newly created evaluator, ready for use. **Throws:**: `java.lang.Exception` - if the evaluator name is invalid, or the options supplied are not acceptable to the evaluator

---


### makeCopies

```
public static ASEvaluation[] makeCopies(ASEvaluation model,
                                        int num)
                                 throws java.lang.Exception
```

:   Creates copies of the current evaluator. Note that this method
    now uses Serialization to perform a deep copy, so the evaluator
    object must be fully Serializable. Any currently built model will
    now be copied as well.

    :   **Parameters:**: `model` - an example evaluator to copy: `num` - the number of evaluator copies to create. **Returns:**: an array of evaluators. **Throws:**: `java.lang.Exception` - if an error occurs

---


### getCapabilities

```
public Capabilities getCapabilities()
```

:   Returns the capabilities of this evaluator.

    :   **Specified by:**: `getCapabilities` in interface `CapabilitiesHandler`
    :   **Returns:**: the capabilities of this evaluator **See Also:**: `Capabilities`

---


### getRevision

```
public java.lang.String getRevision()
```

:   Returns the revision string.

    :   **Specified by:**: `getRevision` in interface `RevisionHandler`
    :   **Returns:**: the revision


---


|  |  |  |
| --- | --- | --- |
| **Overview** \| **Package** \| **Class** \| **Tree** \| **Deprecated** \| **Index** \| **Help** \| **Weka's home** | |  |
| PREV CLASS   **NEXT CLASS** | **FRAMES**    **NO FRAMES**     **All Classes** |  |
| SUMMARY: NESTED \| FIELD \| CONSTR \| METHOD | DETAIL: FIELD \| CONSTR \| METHOD |  |


---
